# Utilizing drugs, food, and natural product libraries: A computational approach to targeting PGAM1 in clinical cancer therapy

**DOI:** 10.1097/MD.0000000000048660

**Published:** 2026-05-08

**Authors:** Abdullah R. Alanzi, Hattan A. Alharbi

**Affiliations:** a Department of Pharmacognosy, College of Pharmacy, King Saud University, Riyadh, Saudi Arabia.

**Keywords:** Cancer, molecular docking, molecular dynamics (MD) simulation, PGAM1, virtual screening

## Abstract

Phosphoglycerate mutase 1 (PGAM1) is an important glycolytic enzyme that plays a significant role in cancer metabolism. It catalyzes the conversion of 3-phosphoglycerate (3-PG) to 2-phosphoglycerate (2-PG), facilitating energy release during glycolysis. PGAM1 is often upregulated in various cancers, including breast, lung, and prostate cancers, contributing to tumor growth and progression by coordinating glycolysis and anabolic processes which makes it an appealing target for drug development. In this study, we screened drugs, food, and natural compound libraries against PGAM1 by using structure based virtual screening approach. A total of 100 compounds from each library were screened and then docked to find the best plausible modes of the hits. Based on the binding affinities, 5 compounds from each library were selected for molecular interactions analysis. After analyzing the interactions, the top compound from each library was subjected to 200 ns simulation to check the effect of hits on the protein. The simulation results revealed that the hits made stable interactions with the protein during simulation and no confirmational changes were observed in the structure of protein. All these findings suggest that the selected compounds can serve as lead compounds in inhibiting the biological activity of PGAM1, but it requires further experimental investigation.

## 1. Introduction

Phosphoglycerate mutase 1 (PGAM1) is an essential enzyme in glycolysis, as it catalyzes the conversion of 3-phosphoglycerate (3-PG) into 2-phosphoglycerate (2-PG), it is an essential part of the metabolic network. Apart from regulating the rate at which glycolysis proceeds and the way sugar is taken up, broken down, and converted into energy inside cancer cells, PGAM1 also regulates the amounts of 3-PG and 2-PG, which therefore affects the functions of other enzymes and metabolic pathways. The expression of PGAM1 is abnormal in many cancers, which promotes the growth and spread of cancer cells and eventually affects the prognosis of malignancy. PGAM1 has thus emerged as a key target in anti-tumor research.^[1–4]^

Currentl, the PGMI-004A, MJE3, and the naturally occurring compound epigallocatechin gallate (EGCG), which is mostly derived from *Camellia sinensis* (green tea) and derivatives of xanthones, are among the few known PGAM1 inhibitors.^[5]^ MJE3 was first discovered to be a PGAM1 inhibitor; however, due to its spiroepoxide substructure, it also covalently modifies the K100 residue of PGAM1 to render it inactive.^[6]^ A proven PGAM1 inhibitor, PGMI-004A has anticancer effects in mice models with tumors xenografted from humans. The described inhibitors’ molecular potencies are both insignificant.^[7]^ Despite having a molecular potency thirty times greater than PGMI-004A, EGCG, PGAM1’s third inhibitor, cannot be regarded as a good inhibitor because to its off-target effects and polyphenol structure.^[6]^ To suppress PGAM1 activity, xanthone derivatives (24 N-xanthone benzene sulfonamides) have recently been developed and synthesized. When compared to PGMI-004A, the majority of them show mild anti-proliferation effects on various cancer cell lines and better effectiveness against PGAM1.^[8,9]^ It is necessary to create and find additional active substances that can bind to the PGAM1 enzyme and limit its metabolic activity because there aren’t many known PGAM1 inhibitors.

Structure-based drug design (SBDD) has become an indispensable approach in modern anticancer drug discovery. By leveraging detailed 3-dimensional structural information of target proteins, SBDD enables the rational design and optimization of therapeutic agents with enhanced specificity and efficacy.^[10]^ Recent studies have demonstrated the successful application of SBDD in identifying potent inhibitors against various cancer-related targets, underscoring its pivotal role in the development of novel anticancer therapies. For instance, the integration of molecular docking and virtual screening techniques has facilitated the discovery of lead compounds with promising anticancer activity, as evidenced by recent research efforts. These advancements highlight the critical importance of SBDD in accelerating the identification and development of effective anticancer agents.^[11]^ Using advanced computational techniques like molecular docking, structure-based virtual screening, and molecular dynamics simulations, we investigated potential PGAM1 inhibitors in this comprehensive study. In addition, we investigate the interactions of identified compounds with drug, food, and natural product libraries to shed light on potential therapeutics.

## 2. Methodology

### 2.1. 3D grid parametrization

The crystallographic structure of PGAM1 was retrieved from PDB (ID: 5Y2I) and prepared for the virtual screening, all extra ligands and crystal waters were removed and the 3D grid was parameterized by AutoDock Tools^[12]^ to find the cartesian coordinates to proceed with the structure-based virtual screening 3D environment for structure-based virtual screening.

### 2.2. Structure-based virtual screening

The prepared structure of the PGAM1 was subjected to virtual screening by using MTiOpenScreen platform (https://bioserv.rpbs.univ-paris-diderot.fr/services/MTiOpenScreen/).^[13]^ This server contains several libraries including natural compound library, drug library, food library etc. MTiOpenScreen ranks the compounds based on the binding affinities, by utilizing Autodock Vina.^[14]^ After conducting screening of 3 libraries i.e., FOOD-lib which is a collection of food-derived compounds aimed at exploring nutraceutical potentials, accessible through MTiOpenScreen for academic purposes, NP-lib which is a natural products library containing diverse compounds from various natural sources, and Drugs-lib which is FDA-approved drugs and is accessible through the MTiOpenScreen platform, which offers free access for academic research, top 100 compounds exhibiting the lowest binding energy values were selected from each library were retrieved and processed using the LigPrep program in Schrödinger’s Maestro.^[15]^ To optimize computational efficiency while focusing on the most promising candidates, this threshold was selected to allow only compounds with the highest potential binding affinities to proceed to rigorous docking and simulation studies. Ligand geometries were refined using the OPLS_2005 force field, which is well-parameterized to ensure energetically favorable conformations; subsequent energy minimization was applied to eliminate unfavorable interactions and relieve structural strain.^[16]^ Energy minimization was applied to remove any unfavorable interactions or strained geometries.

### 2.3. Molecular docking

The screened compounds were subjected to molecular docking against the PGAM1 receptor. The X-ray crystallographic structure of PGAM1 was prepared using the Protein Preparation Wizard.^[17]^ Receptor preparation involved several steps, including the generation of disulfide bonds, assignment of zero-order metal bonds, and addition of hydrogens, while non-essential ligands and crystallographic water molecules were removed. Protonation states of ionizable groups were optimized at physiological pH (7.0) using the PROPKA program,^[18]^ followed by energy minimization with the OPLS_2005 force field. A 3-dimensional receptor grid was then generated at Cartesian coordinates X = -0.08, Y = 36.96, and Z = 26.83 to define the docking site. Finally, the screened ligands were docked into the receptor grid using the Standard Precision (SP) mode of Glide.^[19]^

### 2.4. Molecular dynamics (MD) simulation

Only the top-ranked compound from each library was selected for molecular dynamics (MD) simulations. The protein–ligand complexes were simulated for 200 ns using Desmond^[20]^ to assess protein conformational stability and ligand binding persistence. Each system was solvated in an orthorhombic box with a 10 Å buffer using the TIP3P water model,^[21]^ neutralized with counter ions, and supplemented with 0.15 M NaCl to mimic physiological conditions. Simulations were conducted under an NPT ensemble at 300 K and 1 atm. Following system relaxation, a 200 ns production run was performed, with trajectories saved every 50 ps for subsequent analysis using the Simulation Interaction Diagram module of Desmond.

## 3. Results

### 3.1. 3D grid parametrization

**Figure 1.**
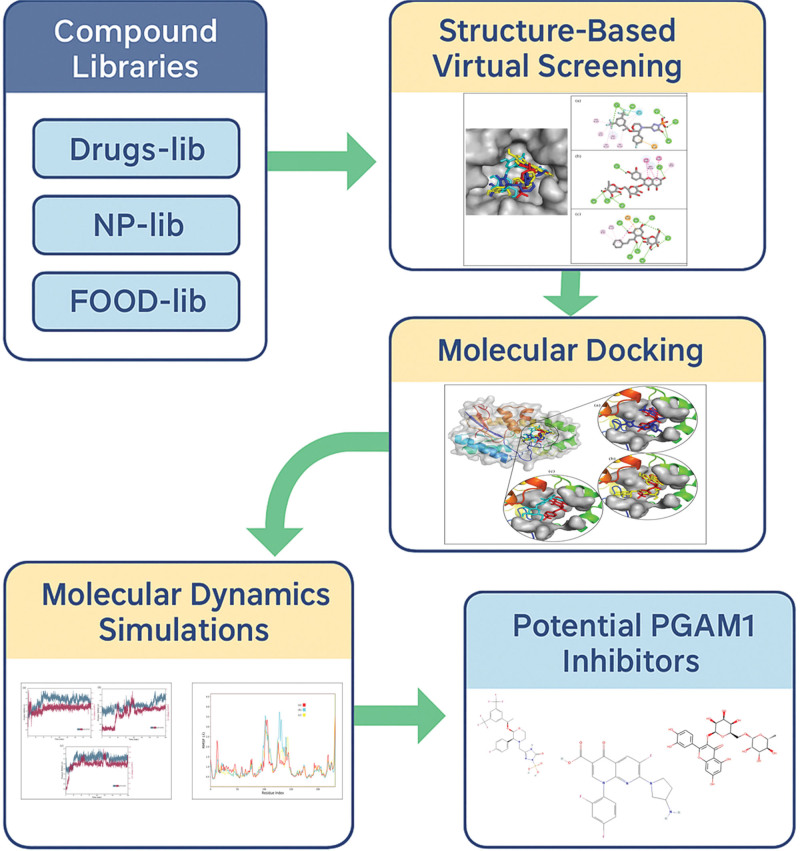
Graphical abstract of the whole study with depicting the flow and the results of the current study.

**Figure 2.**
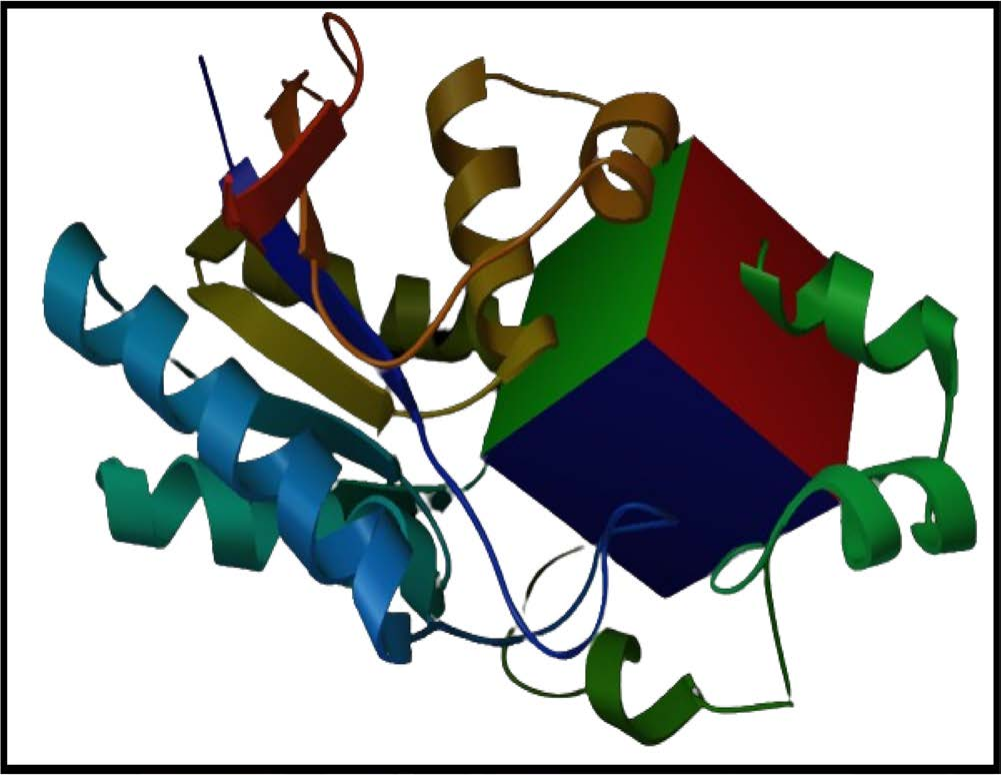
The visual representation of grid box around the binding pocket of PGAM1.

The graphical abstract of the study is presented in Figure 1 to showcase the flow of the study. The residues in the binding pocket of the PGAM1 protein were parameterized within a 3-dimensional grid box. The coordinates for this grid were determined using a co-crystal ligand as a reference. Specifically, the internal coordinates for the grid box were set at −0.08 for x, 36.96 for y, and 26.83 for z. A visual representation of the 3D grid box is illustrated in Figure 2.

### 3.2. Virtual screening

The structure-based virtual screening of the PGAM1 protein was conducted using MTiOpenScreen webserver. During the screening 3 different libraries i.e., FOOD-lib, Drugs-lib, and NP-lib were selected. FOOD-lib contains 26,941 compounds, Drugs-lib has 21,276 compounds while NP-lib contains 1237 compounds. The 100 compounds from each library were selected against PGAM1 protein based on their binding affinities.^[22]^

### 3.3. Molecular docking

The compounds screened by virtual screening were prepared and their docking study was conducted against the PGAM1 protein.^[23]^ The binding affinities (Table 1) of all docked compounds were analyzed and then the top 5 compounds from each library were selected for further analysis (Table 2). The binding affinities from Drugs-lib were in the range of −9.232 to −8.347, for NP-lib compounds were −8.799 to −8.444, and for FOOD-lib compound were in the range of −8.599 to −8.053 kcal/mol. The docking scores of the selected compounds suggested that these have potential for inhibiting the function of the PGAM1 protein.

**Table 1:** The glide scores of the docked compounds against PGAM1 and the parameters that were used in docking studies.

Sr. No.	Compounds	Glide score (kcal/mol)	Grid Centre (X,Y,Z)	Docking mode	Scoring function
Drugs-lib					
1	Fosaprepitant	−8.691	(−0.08, 36.96, 26.83)	Glide SP	OPLS_2005
2	Olcegepant	−8.183	(−0.08, 36.96, 26.83)	Glide SP	OPLS_2005
3	Fostemsavir	−8.127	(−0.08, 36.96, 26.83)	Glide SP	OPLS_2005
4	Tosufloxacin	−8.094	(−0.08, 36.96, 26.83)	Glide SP	OPLS_2005
5	Indacaterol	−7.944	(−0.08, 36.96, 26.83)	Glide SP	OPLS_2005
NP-lib					
1	ZINC000003947429	−8.799	(−0.08, 36.96, 26.83)	Glide SP	OPLS_2005
2	ZINC000253503169	−8.582	(−0.08, 36.96, 26.83)	Glide SP	OPLS_2005
3	ZINC000253503175	−8.524	(−0.08, 36.96, 26.83)	Glide SP	OPLS_2005
4	ZINC000003947430	−8.494	(−0.08, 36.96, 26.83)	Glide SP	OPLS_2005
5	ZINC000003947428	−8.444	(−0.08, 36.96, 26.83)	Glide SP	OPLS_2005
FOOD-lib					
1	FL1	−8.599	(−0.08, 36.96, 26.83)	Glide SP	OPLS_2005
2	FL2	−8.329	(−0.08, 36.96, 26.83)	Glide SP	OPLS_2005
3	FL3	−8.126	(−0.08, 36.96, 26.83)	Glide SP	OPLS_2005
4	FL4	−8.075	(−0.08, 36.96, 26.83)	Glide SP	OPLS_2005
5	FL5	−8.053	(−0.08, 36.96, 26.83)	Glide SP	OPLS_2005

FL1 = 2_4_6_Trihydroxydihydrochalcone_2_glucoside, FL2 = Nicotinic_acid_adenine_dinucleotide, FL3 = Tetragastrin, FL4 = Riboflavine_5_dihydrogen_phosphate, FL5 = Catechin_7_xyloside.

**Table 2:** The molecular interactions of the selected compounds with PGAM1 protein.

Sr.	Compound code	Interactions
Drugs-lib		
1	Fosaprepitant	Conventional Hydrogen Bond: Arg90, Tyr92, Glu19, Glu89, Arg10 Halogen: Arg116 Pi-Cation: Lys100 Alkyl: Pro123, Leu95, trp115, Phe22, Val112
2	Olcegepant	Conventional Hydrogen Bond: Met206, Arg90, Tyr92, Glu89, Ser23, Asn20 Pi-Sigma: Leu95 Alkyl: Arg116, Trp115, Pro123, Phe22
3	Fostemsavir	Conventional Hydrogen Bond: Met206, Arg10, Arg116, Lys100 Carbon Hydrogen Bond: Asn209, Asn20 Alkyl: Phe22, Trp115, Leu95, Pro123
4	Tosufloxacin	Conventional Hydrogen Bond: Glu19, Arg191, Ser118, Arg90 Carbon Hydrogen Bond: Trp115 Alkyl: Phe22, Arg116
5	Indacaterol	Conventional Hydrogen Bond: Glu19, Phe22, Asn209 Alkyl: Arg116, Trp115, Leu95, Val112
NP-lib		
1	ZINC000003947429	Conventional Hydrogen Bond: Arg10, Glu19, Glu89, Met206, Arg90 Alkyl: Phe22, Trp115, Arg116, Leu95
2	ZINC000253503169	Conventional Hydrogen Bond: Glu89, Arg10, Asn20, Lys100, Arg90, Arg116, Met206 Pi-Sigma: Phe22 Alkyl: Leu95, Pro123, Trp115
3	ZINC000253503175	Conventional Hydrogen Bond: Glu19, Arg10, met206, Arg116, Arg90, Lys100, Asn20 Pi-Cation: Arg191 Alkyl: Phe22, Trp115, Tyr92
4	ZINC000003947430	Conventional Hydrogen Bond: Glu89, Arg10, Glu19, Lys100, Asn20 Alkyl: Tyr92, Phe22, Trp115, Arg116
5	ZINC000003947428	Conventional Hydrogen Bond: Arg191, Met206, Arg10, Glu19, Tyr92, Glu89 Alkyl: Phe22, Arg116
FOOD-lib		
1	FL1	Conventional Hydrogen Bond: Arg116, Phe22, Asn209, Glu19, Arg10, Tyr92, Arg90 Pi-Cation: Arg191 Alkyl: Leu95, Pro123
2	FL2	Conventional Hydrogen Bond: Ser23, Glu89, Arg10, Arg90 Carbon Hydrogen Bond: Arg116, Glu19 Alkyl: Phe22
3	FL3	Conventional Hydrogen Bond: Arg116, Tyr92, Asn188, Met206 Carbon Hydrogen Bond: Arg90 Alkyl: Phe22
4	FL4	Conventional Hydrogen Bond: Arg191, Asn209, Met206 Salt Bridge: Arg116, Lys100 Alkyl: Phe22, Tyr92, Trp115, Leu95, Pro123
5	FL5	Conventional Hydrogen Bond: Arg10, Glu19, trp115, Arg191, Arg90 Carbon Hydrogen Bond: Glu89, Tyr92, Arg116 Alkyl: Phe22, Pro123 Van der Waals: Arg116

### 3.4. Post docking analysis

**Figure 3.**
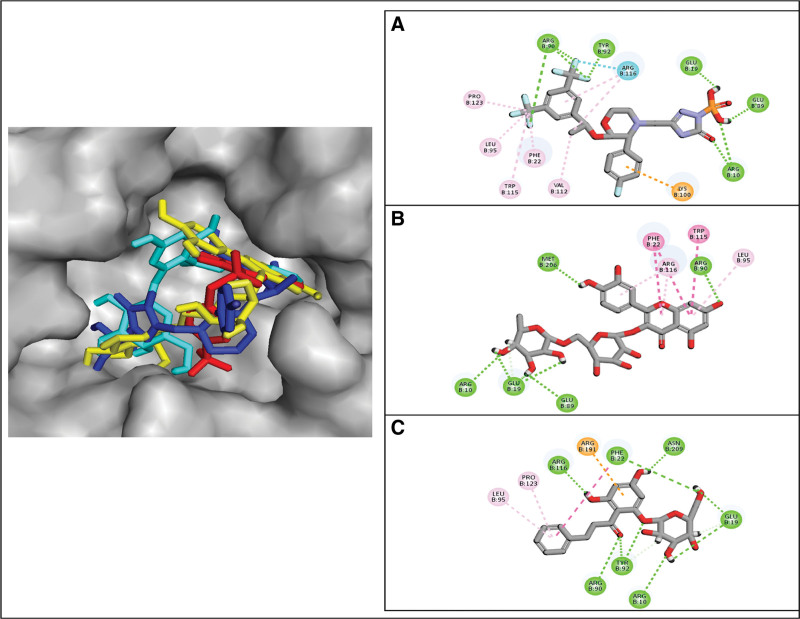
The interactions of the top compound from each library with PGAM1. (A) Fosaprepitant, (B) ZINC000003947429, (C) FL1.

The docked poses of the selected compounds were analyzed by Discovery Studio client tool to find the molecular interactions. The molecular interactions mainly involved hydrogen bonding, van der Waal interactions, pi-pi stacking, pi-sigma interactions, and alkyl (hydrophobic) interactions. The molecular interactions of each candidate compound help in determining the binding affinities. Especially, the hydrogen bonds among ligand and protein atoms play an important role in the strength of protein-ligand complex.^[24]^ In the molecular interaction analysis of compounds selected from Drugs-lib, Fosaprepitant formed 5 conventional hydrogen bonds with Arg90, Tyr92, Glu19, Glu89, Arg10, one halogen interaction with Arg116, one Pi-Cation interaction with lys100, and 5 alkyl interactions with Pro123, Leu95, trp115, Phe22, and Val112 (Fig. 3a). The molecular interactions of other Drugs-lib compounds are show in in Table 2. Similarly, from NP-lib, ZINC000003947429 formed 5 conventional hydrogen bonds with Arg10, Glu19, Glu89, Met206, Arg90, and 4 alkyl interactions with Phe22, Trp115, Arg116, Leu95 (Fig. 3b). Similarly, FL1 from FOOD-lib made 7 conventional hydrogen bonds with Arg116, Phe22, Asn209, Glu19, Arg10, Tyr92, Arg90, one Pi-Cation interaction with Arg191, and 2 alkyl interactions with Leu95, Pro123 (Fig. 3c). The molecular interactions of remaining compounds are shown in Table 2.

### 3.5. Analysis of plausible binding modes

**Figure 4.**
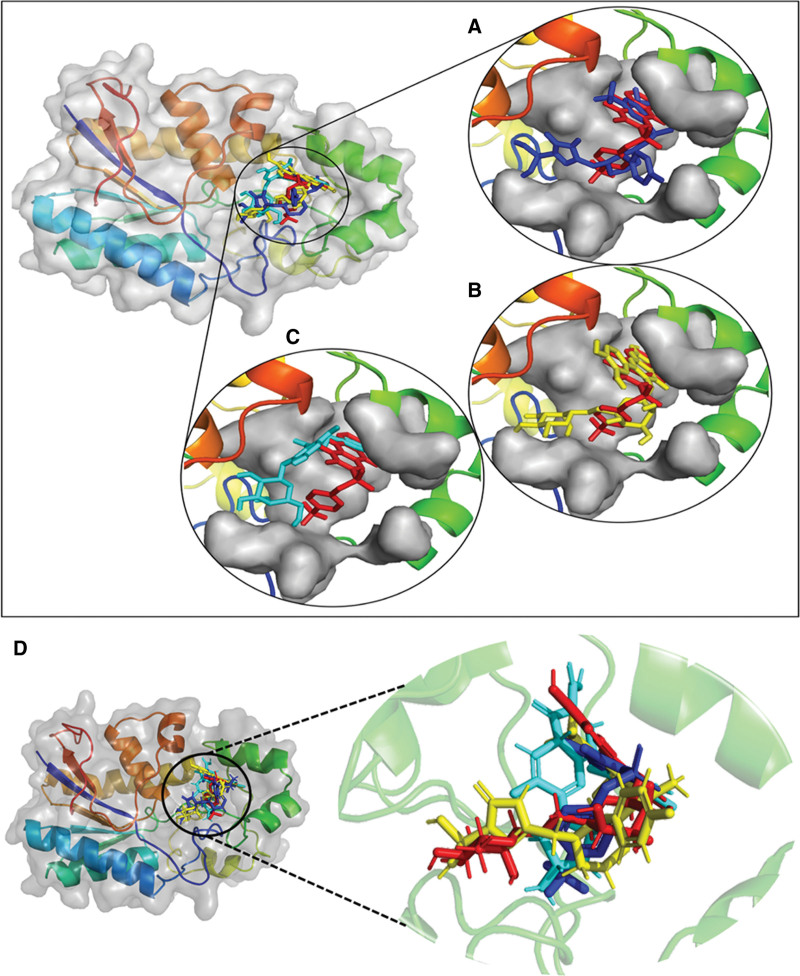
The plausible binding poses of the top 4 compounds (Red sticks: co-crsytal ligand). (A) Fosaprepitant (Blue sticks), (B) ZINC000003947429 (Yellow sticks). (C) FL1 (Cyan sticks). (D) The redocking validation of PGAM1’s inhibitor PGMI-004A (red) with this study’s top inhibitory candidates Fosaprepitant (Blue sticks), ZINC000003947429 (Yellow sticks), and FL1 (Cyan Sticks), all of them turned out to be exhibiting the same binding site with almost same poses.

The plausible binding modes of the selected compounds were observed by alignment on co-crystal ligand. This alignment showed that the docked poses of the hits occupied the same space in binding sites of the PGAM1 as occupied by co-crystal ligand (Fig. 4a-c). As a result, the plausible binding modes of the docked hits were evaluated for stability through MD simulations. To further substantiate the relevance of our docking results, redocking validation of the known inhibitor and adaptation of the strategy described by Huang et al (2019), who identified Phe22, Lys100, Arg116, and Arg191 as critical allosteric residues through site-directed mutagenesis and inhibitor validation was taken in place.^[25]^ Docking poses were examined for the top candidates (e.g., Fosaprepitant, ZINC000003947429, FL1) for interactions (hydrogen bonds, hydrophobic contacts, π interactions) with the reported residues. Notably, Fosaprepitant forms pi-alkyl, pi-cation, halogen bond with Phe22, Lys100, and Arg116, respectively. ZINC000003947429 engages with a pi-pi sacked, pi-alkyl with Phe22, Arg116 respectively. In the case of FL1, Phe22 and Arg116 forms a hydrogen bond, while Arg191 forms a pi-cation bond, supporting the potential involvement of the allosteric site in ligand binding (Fig. 3a-c). Moreover, the known inhibitor was also docked against PGAM1 for a comparative analysis (Fig. 4d) PGMI-004A’s binding affinity was −7.89 kcal/mol. These observations strengthen the mechanistic credibility of our computational screening and align with experimentally validated structural determinants of PGAM1 inhibition.

### 3.6. MD simulation

#### 3.6.1. RMSD

**Figure 5.**
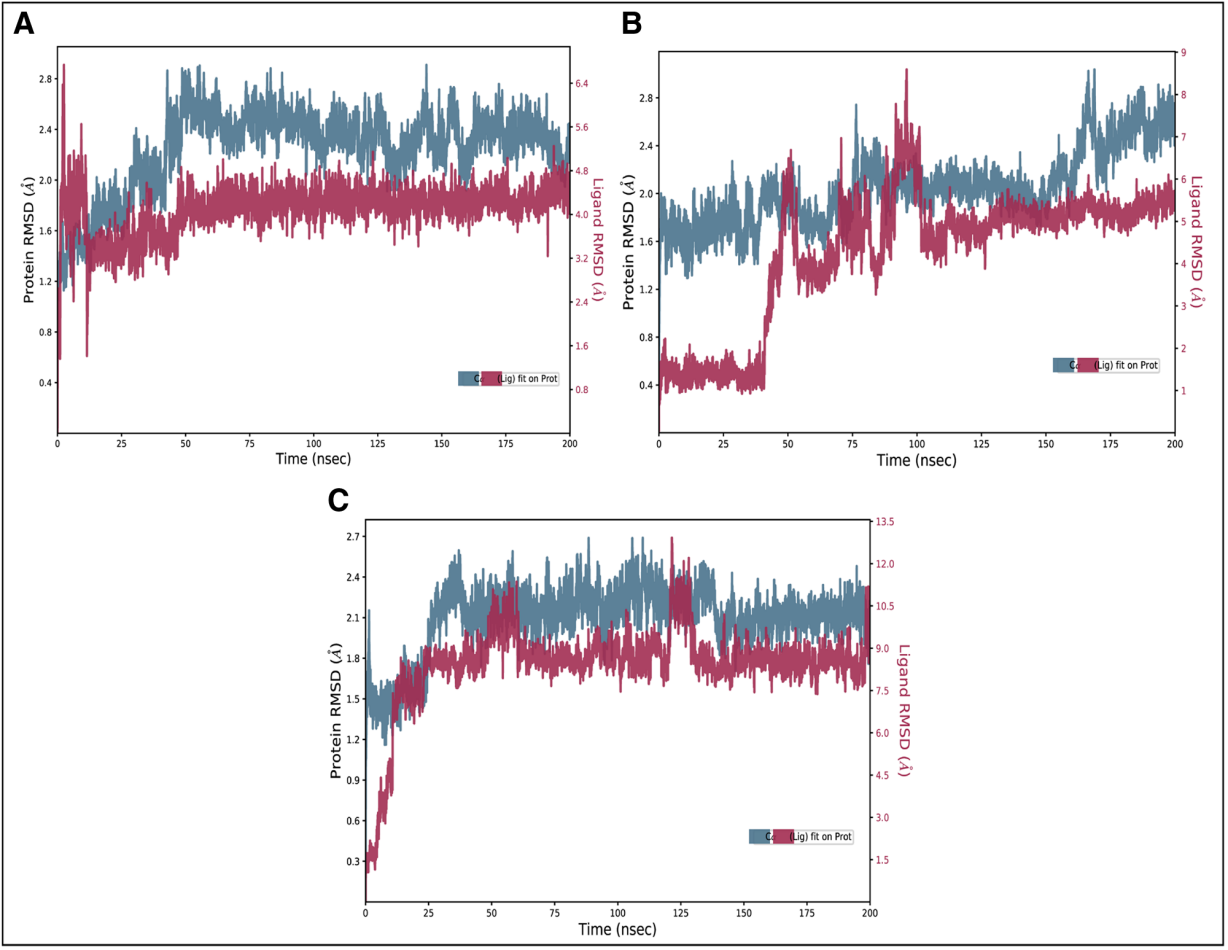
The RMSD plots of the PGAM1 complexes. (A) Fosaprepitant, (B) ZINC000003947429, (C) FL1.

The root mean square deviation of the Cα atoms was measured to evaluate the structural changes of the complexes.^[26,27]^ The RMSD values for the Fosaprepitant complex gradually increased to 2.4 Å at 50 ns and then maintained this range till the end of simulation while the RMSD of ligand fit was slightly lower than the protein RMSD (Fig. 5a). The RMSD of NP compound ZINC000003947429 remained in the range of 2 Å till 150 ns and then showed minor deviations of 0.4 Å towards the end of simulation while the RMSD of ligand fit was lower than protein in first half but after 100 ns, it was aligned on the protein RMSD (Fig. 5b). Lastly, the RMSD of FL1 complex showed stable trend after 25 ns at 2.4 Å and the ligand RMSD was perfectly aligned on the protein RMSD (Fig. 5c).

#### 3.6.2. RMSF

**Figure 6.**
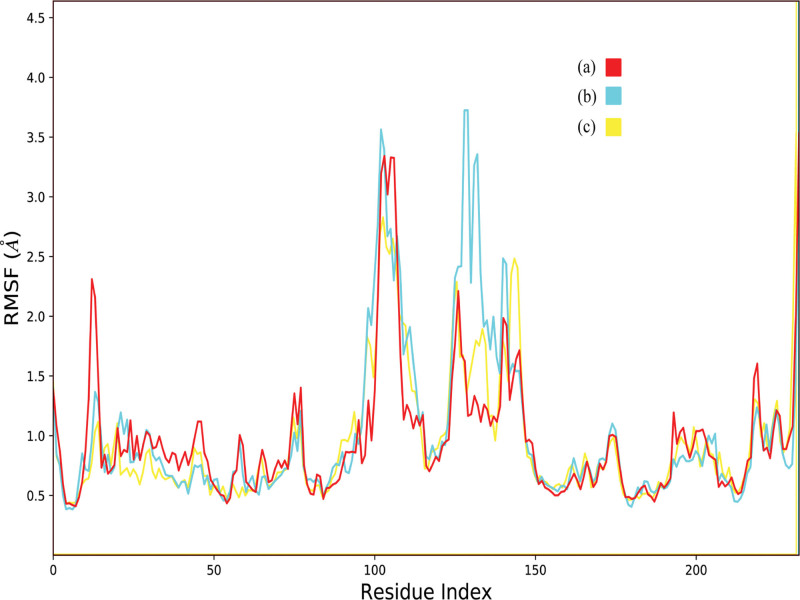
The comparative RMSF plots of the PGAM1 protein in the presence of hit compounds. (A) Fosaprepitant, (B) ZINC000003947429, (C) FL1.

RMSF values were calculated to investigate the protein residues dynamics upon interacting with the ligands.^[28]^ The RMSF analysis showed that most protein residues remained stable during the simulations, with values below 2 Å, indicating that ligand binding did not induce significant fluctuations in the overall protein structure. In contrast, residues located in loop regions displayed higher mobility in the presence of the hit compounds compared with the co-crystal ligand, with fluctuations reaching up to ~4 Å, suggesting enhanced flexibility in these segments (Fig. 6). Notably, all hit complexes exhibited comparable RMSF profiles, except for ZINC000003947429, which induced additional fluctuations in residues 130–140.

**Figure 7.**
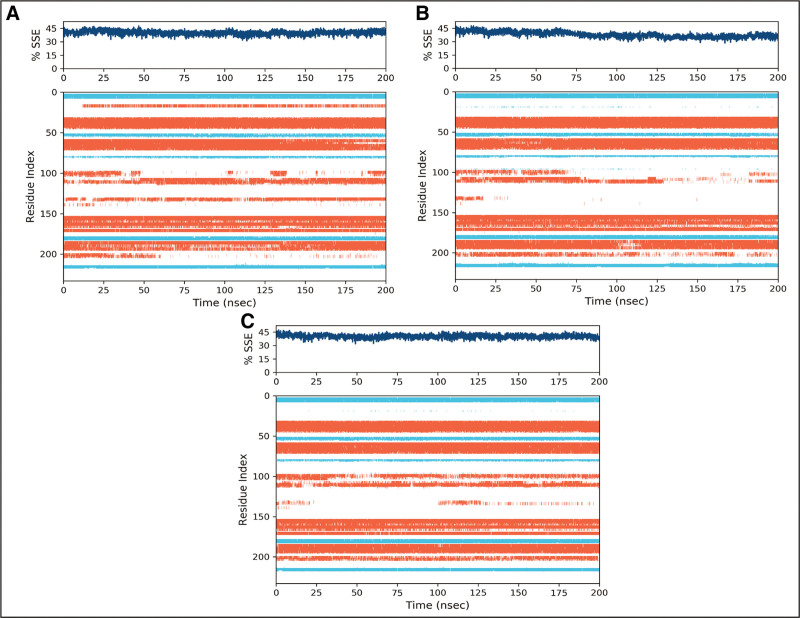
The percentage of secondary structure elements of PGAM1 receptor upon binding of hits. (A) Fosaprepitant, (B) ZINC000003947429, (C) FL1.

Most protein residues exhibited only minor changes, while the loop regions displayed considerably greater flexibility (Fig. 7). The blue regions showed the presence of alpha helices while orange color indicates the beta sheets. The loops were exhibited in white color. During the simulation, it was estimated that the secondary structures did not show fluctuations and remained stable upon binding of the ligands.

#### 3.6.3. Protein-ligand contacts

**Figure 8.**
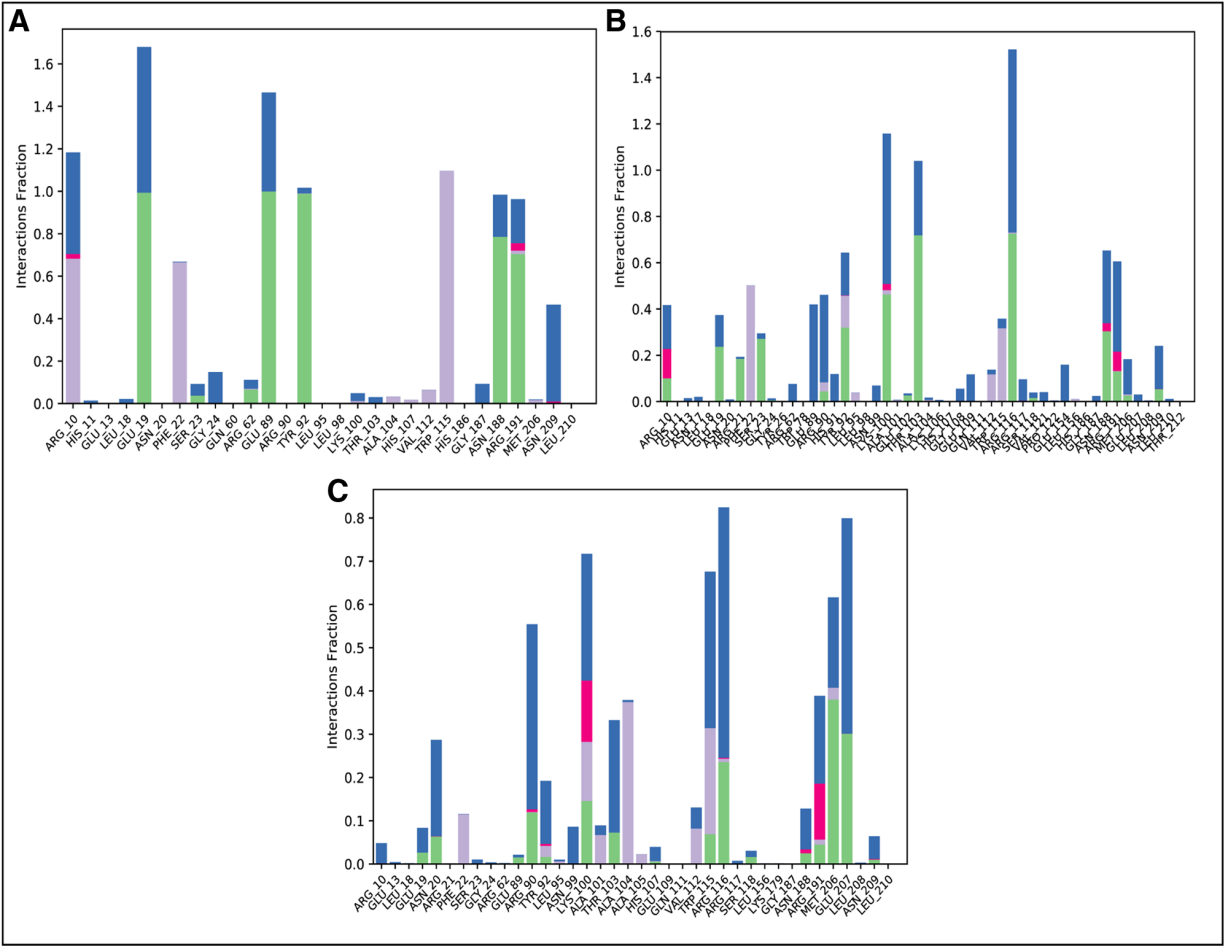
The protein-ligand interactions. (A) Fosaprepitant, (B) ZINC000003947429, (C) FL1. Green bars show hydrogen bonding, gray show hydrophobic interactions, and blue shows the water bridges.

During the simulations, protein-ligand interactions were characterized by hydrogen bonding, water bridges, ionic contacts, and hydrophobic interactions, all of which contributed to complex stability. Fosaprepitant formed hydrogen bonds with Glu19, Ser23, Arg62, Tyr92, Asn188, and Arg191 (Fig. 8a). In the ZINC000003947429 complex, hydrogen bonding was observed with Arg10, Glu19, Arg21, Ser23, Tyr92, Lys100, Thr103, Arg116, Asn188, Arg191, and Asn209 (Fig. 8b). Similarly, FL1 established hydrogen bonds with Glu19, Asn20, Arg90, Lys100, Thr103, Trp115, Arg116, Ser118, Asn188, Arg191, Met206, Glu207, and Asn209 (Fig. 8c). These persistent hydrogen bonding patterns highlight the involvement of specific residues in stabilizing the complexes, thereby strengthening binding affinity and supporting the conformational stability observed in MD simulations.^[29]^

#### 3.6.4. Hydrogen bonding

**Figure 9.**
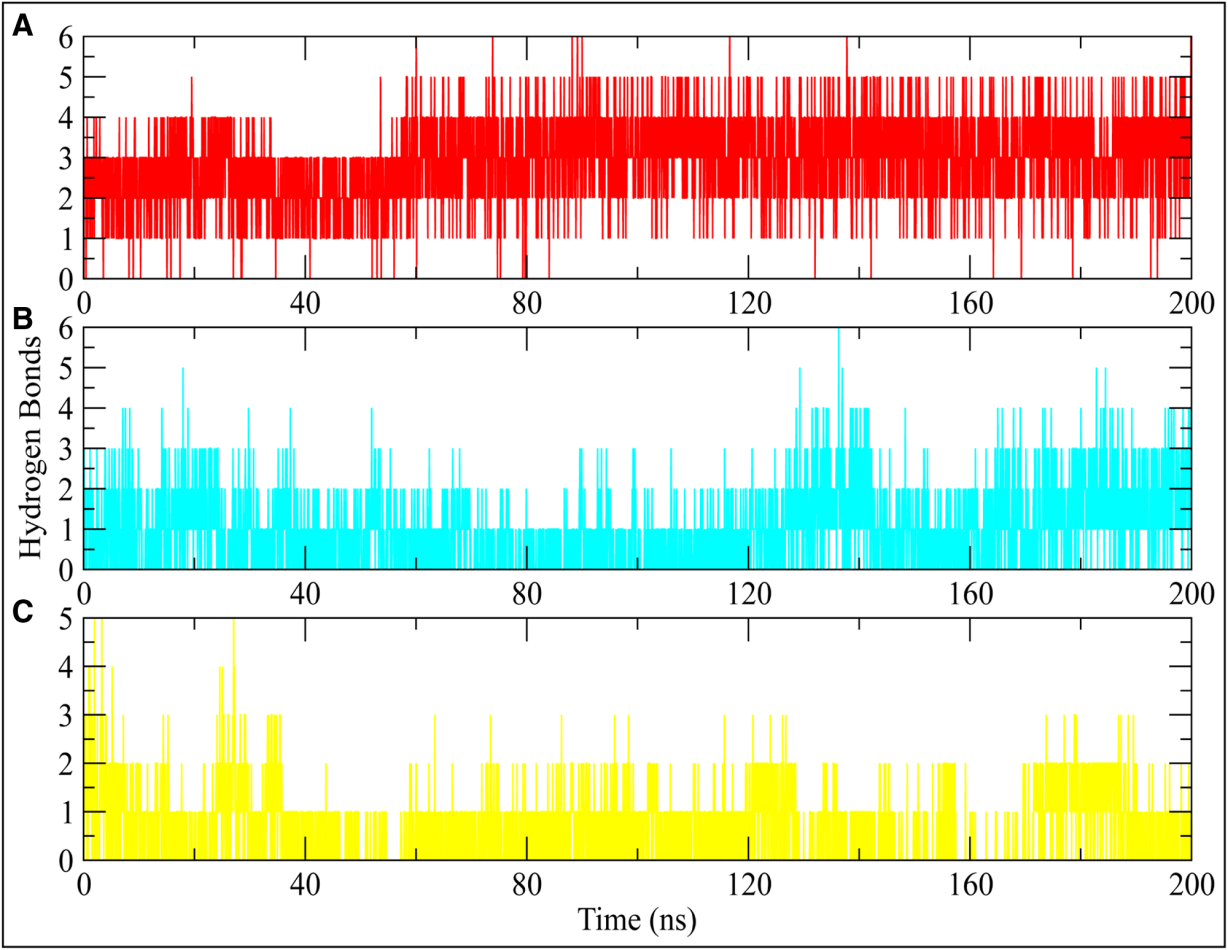
The hydrogen bonding between hits and PGAM1 protein. (A) Fosaprepitant, (B) ZINC000003947429, (C) FL1.

Hydrogen bonding is a critical factor for the stability of protein-ligand complexes.^[30]^ Throughout the simulation, the number of hydrogen bonds formed between the ligand and the active site residues was analyzed. The hydrogen bonding plots indicate that Fosaprepitant consistently formed at least 4 hydrogen bonds during the simulation, with some frames showing 5 and others displaying 6 hydrogen bonds (Fig. 9a). In contrast, ZINC000003947429 formed at least 3 hydrogen bonds throughout the simulation (Fig. 9b). Meanwhile, FL1 maintained a minimum of 2 hydrogen bonds during the simulation (Fig. 9c).

## 4. Discussion

One of the main enzymes in glycolysis, the main route for energy metabolism in cells, is phosphoglycerate mutase 1 (PGAM1). Because cancer cells typically exhibit dysregulation of PGAM1, an essential glycolysis enzyme, it is an attractive target for therapeutic intervention. Identifying PGAM1 inhibitors is critical not only for improving our understanding of cellular metabolism, but also for developing potential treatments that can selectively target cancer cells while having minimal impact on normal, healthy cells.^[2,31,32]^ The study of PGAM1 inhibitors using computational methods is a valuable starting point for drug discovery efforts aimed at combating diseases associated with altered glycolytic pathways, particularly cancer. Several classes of small-molecule inhibitors have been reported to date; these differ in scaffold, mechanism of inhibition (active-site competitive, covalent modification, or allosteric modulation), potency, and drug-like properties. Summarizing these distinct chemotypes and mechanisms helps place the current in-silico screening results in context and clarifies opportunities and limitations for lead optimization. In this study, we use advanced computational methods to identify potential PGAM1 inhibitors and investigate how they interact with various compound libraries.

The selection of PDB ID: 5Y2I for virtual screening study was based on its high-resolution (1.92 Å) X-ray diffraction data, complexed with the inhibitor PGMI-004A, offering valuable insights into the active site conformation and key interactions critical for inhibitor binding. The protein originates from *Homo sapiens*, ensuring that the findings are directly applicable to human biology and potential therapeutic applications. The structure is a homo-dimer, representing the biologically relevant form of PGAM1, which is important for understanding its functional dynamics. The crystal structure of PGAM1 was prepared, and the binding pocket residues were parameterized within a 3-dimensional grid box. Structure-based virtual screening of PGAM1 was conducted using the Drugs-lib, NP-lib, and FOOD-lib databases, focusing on binding affinities. A total of 100 compounds from each library were screened against PGAM1. The hit compounds identified during the virtual screening were then docked to the prepared PGAM1 receptor using the Glide tool’s standard precision mode to predict their binding affinities. The OPLS_2005 force field was used for energy minimization because of its robust and validated parameterization for small molecules and biological macromolecules. It provides accurate modeling of intramolecular and intermolecular interactions, thereby ensuring reliable optimization of protein-ligand complexes. Additionally, OPLS_2005 is specifically compatible with Schrödinger’s Maestro and Glide modules, making it an ideal choice for structure-based virtual screening and docking workflows. This step is crucial for assessing the potential efficacy of the compounds in inhibiting PGAM1 enzymatic activity.^[33]^ The top 5 compounds from each library were selected for further analysis based on their binding affinities. The binding affinities for the compounds from the Drugs-lib ranged from -9.232 to −8.347 kcal/mol, while those from the NP-lib ranged from −8.799 to −8.444 kcal/mol, and the FOOD-lib compounds exhibited affinities between −8.599 and −8.053 kcal/mol.

The molecular interactions of the selected hit compounds with the binding pocket of the PGAM1 receptor were analyzed. The most frequently observed interactions included conventional hydrogen bonds, carbon-hydrogen bonds, van der Waals interactions, Pi-Sulfur interactions, Pi-Pi stacking, Pi-Sigma interactions, and alkyl interactions. These interactions are crucial for determining the binding affinities and docking scores of the top candidate compounds.

Following the analysis of these molecular interactions, the leading compounds from each library were aligned with co-crystal ligands to evaluate their potential binding poses. The analysis indicated that the docked compounds aligned closely with the co-crystal ligand, exhibiting similar binding modes.

The dynamic behavior of PGAM1 inhibitor complexes over time was examined using MD simulations. This computational approach allows researchers to investigate the flexibility and stability of binding interactions, providing valuable insights into structural changes that may influence the effectiveness of the inhibitors.^[34]^ The MD simulations demonstrated that these compounds remained effective inhibitors within the protein’s binding region. Overall, these findings suggest that the identified hit compounds have the potential to serve as lead compounds for inhibiting PGAM1’s biological activity.

Early PGAM1 inhibitors included electrophilic covalent probes such as MJE3, discovered by chemoproteomic phenotypic screening; MJE3 covalently labels the active-site Lys100 and inactivates the enzyme in cells, demonstrating that targeting this residue can block enzyme function but also raising potential selectivity and safety concerns associated with covalent electrophiles.^[5]^ In parallel, PGMI-004A was developed as a non-covalent small-molecule probe that validated the concept of PGAM1 inhibition in cellular and xenograft models and showed in vivo antitumor activity, albeit at moderate molecular potency relative to later scaffolds.^[1]^ Natural products have also been providing useful scaffolds for PGAM1 inhibition. (−)-Epigallocatechin-3-gallate (EGCG), a green-tea polyphenol, was identified by biochemical screening as a relatively potent PGAM1 inhibitor in vitro (IC_50_ ≈ 0.49 μM) and was shown to bind PGAM1 and modulate intracellular 2-PG levels; however, EGCG’s high polarity and multiple phenolic groups reduce membrane permeability and raise concerns about promiscuous/polypharmacologic effects and bioavailability without formulation or derivatization.^[6]^ A notable conceptual advance for PGAM1 inhibition has been the discovery of allosteric inhibitors that bind outside the canonical substrate pocket and prevent catalysis or block conformational changes required for activity. The Cell Metabolism study that reported HKB99 (and related allosteric compounds) demonstrated that allosteric blockade can impair PGAM1’s catalytic cycle and non-metabolic interactions (e.g., protein-protein interactions), and produced strong cellular and in vivo anti-tumor effects illustrating that allosteric mechanisms may yield improved selectivity and functional outcomes compared with competitive inhibitors.^[25]^

Overall, these diverse inhibitor classes highlight several lessons relevant to our screening results. In light of these considerations, the present virtual screening results should be interpreted as identification of lead scaffolds rather than final drug candidates. We therefore supplemented our docking and MD findings with residue-centric analysis (focusing on Phe22, Lys100, Arg116 and Arg191) and redocking/validation steps (see Figure 5) For future work, prioritized experimental validation (biochemical IC_50_, SPR/ITC binding measurements, site-directed mutagenesis of identified residues, and cell-based metabolic assays) will be necessary to confirm mechanism, on-target activity, and cellular efficacy.

## 5. Conclusion

This study on PGAM1 inhibition demonstrates the power of computational methods in drug discovery. The identified inhibitors show promise for future experimental validation, emphasizing the importance of combining *in silico* approaches with drug, food, and natural product interactions. The findings of this study add to the ongoing efforts to develop targeted therapies for diseases associated with dysregulated glycolysis, particularly cancer. While our structure-based virtual screening and MD simulations identify plausible lead scaffolds, we acknowledge the limitations of purely in-silico ranking. Experimental validation (enzyme IC_50_, binding assays such as SPR/ITC, and cellular metabolic readouts) together with orthogonal computational re-ranking (MM-GBSA/ per-residue decomposition, ensemble docking, and FEP where feasible) are required to confirm potency, binding mode, and mechanism. These steps will be required before any medicinal chemistry optimization or preclinical testing.

## Acknowledgments

Authors would like to express their appreciation to Ongoing Research Funding program, (ORF-2026-885) at King Saud University, Riyadh, Saudi Arabia for supporting this research.

## Author contributions

**Conceptualization:** Abdullah R. Alanzi.

**Data curation:** Hattan A. Alharbi.

**Formal analysis:** Hattan A. Alharbi.

**Investigation:** Abdullah R. Alanzi.

**Methodology:** Hattan A. Alharbi.

**Supervision:** Abdullah R. Alanzi.

**Validation:** Abdullah R. Alanzi.

**Writing – original draft:** Hattan A. Alharbi.

**Writing – review & editing:** Abdullah R. Alanzi.
